# Manganese-Enhanced Magnetic Resonance Imaging: Overview and Central Nervous System Applications With a Focus on Neurodegeneration

**DOI:** 10.3389/fnagi.2018.00403

**Published:** 2018-12-13

**Authors:** Ryan A. Cloyd, Shon A. Koren, Jose F. Abisambra

**Affiliations:** ^1^Department of Physiology, University of Kentucky, Lexington, KY, United States; ^2^College of Medicine, University of Kentucky, Lexington, KY, United States; ^3^Sanders-Brown Center on Aging, University of Kentucky, Lexington, KY, United States; ^4^Department of Neuroscience & Center for Translational Research in Neurodegenerative Disease, University of Florida, Gainesville, FL, United States; ^5^Spinal Cord and Brain Injury Research Center, University of Kentucky, Lexington, KY, United States

**Keywords:** manganese, MEMRI, MRI, mangafodipir, CNS imaging

## Abstract

Manganese-enhanced magnetic resonance imaging (MEMRI) rose to prominence in the 1990s as a sensitive approach to high contrast imaging. Following the discovery of manganese conductance through calcium-permeable channels, MEMRI applications expanded to include functional imaging in the central nervous system (CNS) and other body systems. MEMRI has since been employed in the investigation of physiology in many animal models and in humans. Here, we review historical perspectives that follow the evolution of applied MRI research into MEMRI with particular focus on its potential toxicity. Furthermore, we discuss the more current *in vivo* investigative uses of MEMRI in CNS investigations and the brief but decorated clinical usage of chelated manganese compound mangafodipir in humans.

## Introduction

The use of manganese and similar paramagnetic contrast agents began shortly after the development of magnetic resonance imaging (MRI). Manganese (II) chloride is the most commonly utilized manganese species for manganese-enhanced MRI (MEMRI). Though MEMRI has been widely employed in imaging studies of various investigative directions, the primary focus of this review will be the role of functional MEMRI in the nervous system beginning with a brief historical introduction.

### History

Some of the earliest work into MRI was performed by Paul Lauterbur in 1973 (Lauterbur, 1973). This work contributed to the basis of nuclear magnetic resonance (NMR) and MRI studies as they exist today and resulted in Lauterbur receiving the 2003 Nobel Prize in Physiology and Medicine with Peter Mansfield. Lauterbur et al. (1980) showed that manganese enhanced magnetic images by shortening proton relaxation time, and soon manganese contrast grew into a common imaging staple in living systems. Early uses of manganese contrast were aimed at delineating normal and abnormal tissue. For example, early studies used manganese MRI to study ischemic myocardium in dogs (Brady et al., 1982; Goldman et al., 1982), cerebral edema in cats (Shirakuni et al., 1985) and human tumor xenografts in mice (Ogan et al., 1987). The discovery of paramagnetic enhancement in MRI also lead to research into other paramagnetic contrast agents including gadolinium (Couet et al., 1984; Runge et al., 1984; Fornasiero et al., 1987).

Lin and Koretsky (1997) first demonstrated manganese contrast can be used as a noninvasive, direct measurement of neuronal function. Lin and Koretsky (1997) administered manganese chloride via a peripheral intravenous injection and reported imaging enhancement in stimulated brain regions not affected by changes in blood flow, strongly supporting MEMRI as a direct functional imaging measure. The major advantage of Lin and Koretsky’s (1997) novel application of manganese enhancement was the ability to measure neuronal function *in vivo*. However, this initial application of MEMRI was limited by the need to co-administer mannitol to disrupt the blood-brain barrier. Subsequent studies have refined MEMRI to visualize neuronal activity. A recent study demonstrated that with a sufficiently strong magnetic field (17.1T), MEMRI can be used to visualize action potentials in individual Aplysia buccal neurons (Svehla et al., 2018). Following the discovery that radioactive manganese transports along neural tracts in a microtubule-dependent fashion (Sloot and Gramsbergen, 1994), Koretsky’s group used manganese as a non-radioactive neuronal connection tracer (Pautler et al., 1998). Manganese-enhanced tract tracing has since been used in conjunction with techniques such as diffusion tensor imaging (DTI) to study brain region connectivity and validate tractography studies (Lin et al., 2001; Knosche et al., 2015).

Today, MEMRI is used in three major types of MRI protocols: anatomic studies, functional studies and tractography studies. In the case of anatomic studies, manganese functions much like gadolinium or other paramagnetic contrast agents, and such studies will not be a major focus of this review. In contrast, unique properties of manganese compared to other paramagnetic agents (which will be discussed in more detail in section “Pharmacodynamics” of this review) allow MEMRI to provide information about the function and connectivity of brain regions. Specific instances of these studies will be discussed in regard to specific fields of study in section “MEMRI in CNS imaging” of this review. The ability to perform anatomical, functional and connectivity studies with a single technique has allowed MEMRI to be used to describe dynamic systems *in vivo*. A series of studies from the Van der Linden group used several different types of manganese enhanced MRI protocols to describe the song generation and control in songbirds (Van der Linden et al., 2002; Tindemans et al., 2003, 2006; Van Meir et al., 2004). This demonstrates how all three major types of MEMRI applications can be used to study a single topic. Van der Linden et al. (2004) group developed a technique to allow long term study of a single anatomical region (repeated dynamic MEMRI) through the use of a permanent cannula.

These types of studies can be performed via other types of MRI experiments such as blood-oxygen level dependent (BOLD) contrast or DTI/diffusion kurtosis imaging (DKI). While these types of studies are more widely used, they are less direct measurements of activity/connectivity in the brain than MEMRI. MEMRI should not be seen as a replacement for other types of MRI studies but rather as another tool to provide a more complete understanding of *in vivo* brain function. One study that compared functional measurements obtained via BOLD imaging and MEMRI found that the techniques produce consistent results, demonstrating the potential for them to be used in conjunction (Duong et al., 2000).

### MRI Background

**Figure 1:**
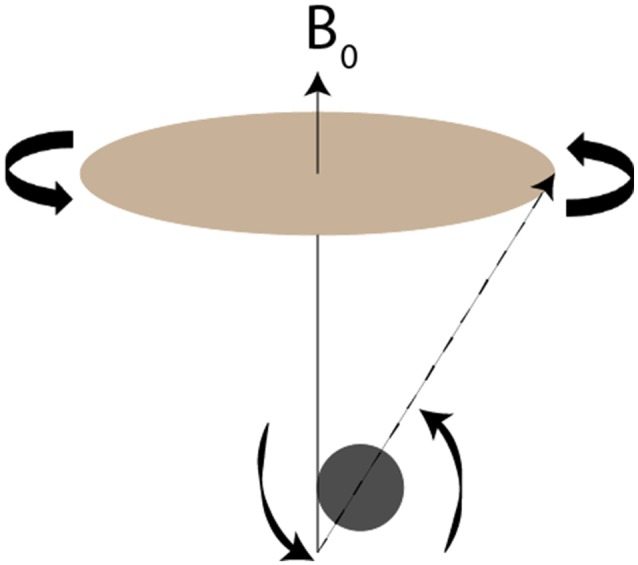
Visual demonstration of precession. Redrawn from Figure 2 of Jackson et al. (2005). The nucleus’s axis of rotation (dotted line) does not exactly align with the magnetic field B_0_ (solid line). As the nucleus rotates around the B_0_, it also spins on its own axis. The frequency with which the nucleus rotates around B_0_, termed the precession, is inherent to each species and scales linearly with the strength of the magnetic field.

A basic understanding of the principles underlying manganese and other paramagnetic contrast agents aids in understanding their enhancement of magnetic images. MRI depends on the spin, charge and magnetism of specific atomic nuclei, particularly ^1^H but also ^31^P, ^23^Na, ^19^F and ^13^C (Jackson et al., 2005). Application of an external magnetic field reorients these species’ axes of spin approximately with the axis of the field. Each species has a unique frequency of rotation around the magnetic field, termed precession (Figure 1). Since precession is linearly proportional to the strength of the magnetic field, stronger applied magnetic fields produce greater precession, resulting in higher signal-to-noise ratios (SNR). For this reason, the strength of the magnetic field defines the resolution of MR imaging.

Under the main magnetic field force (B_0_) and a secondary set of field gradients, each nucleus rotates so it reorients itself with the field, adopting either an aligned or anti-aligned state (Jackson et al., 2005). Most nuclei reside in the lower energy aligned state with a smaller proportion populating the high energy anti-aligned state. The difference in these populations is the basis of NMR. These aligned and anti-aligned systems absorb electromagnetic energy from a third magnetic force (the radiofrequency (RF) pulse), which briefly equalizes the two nuclear alignment states. As the applied perturbation resolves, the energy emitted is detected by a receiver coil and interpreted to generate the MRI. The gradient fields and RF pulse can be altered to suit the needs of the current experiment. A single scan protocol includes repeated RF pulses in rapid succession.

A major variable measured in MR experiments is relaxation time, defined as the time required to reestablish equilibrium between alignment states. Relaxation time is divided into the spin-lattice (T_1_) relaxation time and the spin-spin (T_2_) relaxation time. T_1_ represents the time required for the axis of the nucleus to realign with the main field in the z-direction. A sample with a longer T_1_ time requires a slower rate of RF pulses to allow for recovery between pulses. T_2_ is based on changes in the rates of rotation that occur following termination of the RF pulse. When nuclei are aligned in the same plane, they initially rotate in phase with each other. After the pulse is removed, the nuclei begin rotating at different rates and the amount of time required for the nuclei to lose phase is the T_2_ relaxation time (Jackson et al., 2005). A single scan protocol measures either T1 or T2. In either case, the signal intensity of the final image is determined by the relaxation time. Both relaxation times are determined by a number of intrinsic and environmental factors, and of particular note, the T_1_ is influenced by the presence of paramagnetic species as discovered by Paul Lauterbur’s group in 1980. Paramagnetic agents cause the nuclei to realign more rapidly resulting in shortening of the T_1_ time, which increases the signal intensity on MR images (Mendonça-Dias et al., 1983).

### MEMRI vs. BOLD

Currently, the most common method used for function MR imaging is BOLD. BOLD uses paramagnetic deoxygenated hemoglobin as a natural contrast agent to measure changes in cerebral blood oxygenation (Ogawa et al., 1990). Oxygenated hemoglobin is non-paramagnetic; therefore, under normoxic conditions, the arterial flow does not contribute to the MR signal acquired by BOLD imaging. Under normal conditions, essentially all of the deoxyhemoglobin in the venous circulation is generated by local tissue metabolism. As a result, BOLD signal provides a measure of total metabolism of brain regions.

While both BOLD and MEMRI allow functional MRI, each technique has strengths and weaknesses that must be considered when designing experiments. One major advantage of BOLD over other types of contrast-enhanced MRI protocols is that it does not require administration of exogenous contrast agents (Ogawa et al., 1990). It provides a rapid assessment of regional and global brain metabolism without exposing the patient or animal to potentially harmful contrast agents. Given concerns over the potential toxicity of chronic manganese exposure (discussed more in section “Toxicity”), BOLD may be preferable for long-term studies requiring repeated exposure to manganese.

The BOLD signal is an aggregate of the metabolism of all the cells in the region and therefore the relative contributions of neurons cannot be distinguished from that of glia or other cells. Furthermore, BOLD implementation is complicated during conditions of generalized hypoxia in the area of interest because of the presence of paramagnetic deoxyhemoglobin in the arterial blood supply (Michaely et al., 2012; Taylor et al., 2015; Wang et al., 2017). Similarly, disruptions in regional hemodynamics caused by tumors or arteriovenous malformations can produce artifacts on BOLD (Zaca et al., 2014). In contrast, manganese enhancement is much more specific for neuronal activity and the signal is less susceptible to contributions from non-neuron cells (discussed in section “Mechanism of Entry and Dispersion of Manganese”). Whereas BOLD indirectly measures brain activity through changes in metabolism, MEMRI directly measures activity through changes in calcium dynamics.

## Pharmacodynamics

As with any contrast agent, manganese is influenced and limited by how the body alters it (pharmacokinetics) and how it alters the body (pharmacodynamics). The pharmacokinetic properties of manganese were recently reviewed elsewhere (Chen et al., 2018). To understand the toxic limitations of manganese, potential administration routes into the body, and downstream applications, it is critical to first understand the biological mechanism of action and transport of manganese.

### Mechanism of Entry and Dispersion of Manganese

Out of all paramagnetic contrast agents used as MRI contrasts, manganese has unique application capabilities based on its ability to form a divalent cation with an ionic radius similar to that of calcium. The ability of manganese ions to impede calcium transportation has been recognized since the 1960s (Hubbard et al., 1968), although the precise mechanism (now known to be due to competition for transport) would not be recognized until later. Understanding of the biological mechanisms of manganese developed in conjunction with advances in its uses for imaging purposes, beginning in the early 1980s when its accumulation (Hunter et al., 1980), permeability (Ribalet and Beigelman, 1980) and calcium channel competition (in cardiac tissue; Payet et al., 1980), in nerve terminals (Kita et al., 1981) was discovered. The passage of manganese ions through calcium channels was further supported by the prevention of Mn^2+^-induced changes in nerve terminal activity caused by administration of the calcium channel blockers verapamil (Narita et al., 1990) and later diltiazem, which was found to suppress MEMRI changes following forepaw stimulation in rats (Lu et al., 2007). These studies by Narita et al. (1990) and Lu et al. (2007) as well as others (Carlson et al., 1994) support the hypothesis that the primary entry point for manganese into neurons is through L-type calcium channels; though other studies from as early as 1987 (Mayer and Westbrook, 1987) show evidence manganese may also transverse through other channel types such as NMDA and AMPA receptors.

For example, Itoh et al. (2008) studied the effects of NMDA modulation on MEMRI signal and found drug-induced activation of NMDA receptors increased signal intensity while non-competitive antagonism of the receptors reduced signal intensity, suggesting NMDA receptors play a role in facilitating manganese transport. They found no changes associated with AMPA modulation. A later study by Hankir et al. (2012) further supported the hypothesis that manganese can pass through NMDA receptors, with contrasting evidence suggesting that AMPA receptors mediate manganese enhancement in certain brain structures. However, the two studies used substantially different dosages of AMPA receptor antagonist NBQX (Hankir et al., 2012 used a dose of approximately 40 mg/kg compared to the 10 mg/kg dose used by Itoh et al., 2008), possibly accounting for differences between the two studies. Given this difference, it seems plausible that AMPA receptors do contribute to the transport of manganese through the blood brain barrier, but the role is smaller than that of the NMDARs. Recent work has supported the role of NMDARs in controlling blood brain barrier permeability (Vazana et al., 2016), however the reliance on NMDA of manganese penetrance into the brain was not studied. Though the exact mechanism of calcium channel entry of manganese into the brain is not understood, it is this capacity that facilitates the usage of manganese as a more direct functional imaging method in MEMRI.

Recently, the Turnbull group showed that manganese uptake is also mediated by the divalent metal transporter, DMT1 (Bartelle et al., 2013). By inducing DMT1 expression, Bartelle et al. (2013) achieved MEMRI signal in cell populations (human embryonic kidney, glioma and melanoma) that would not normally be susceptible to manganese enhancement. After this finding, Turnbull’s group induced expression of the bacterial manganese-binding protein MntR in mammalian cells to increase signal enhancement (Bartelle et al., 2015). Expression of MntR, which can be targeted to the Golgi apparatus, endoplasmic reticulum, or cytosol, increases intracellular manganese concentration by preventing efflux of manganese from cells. This paradigm allows for any tissue type to potentially be specifically enhanced via MEMRI. For example, transplanted cells expressing DMT1 can be effectively tracked via MEMRI (Lewis et al., 2015). Future development of the DMT1 MRI reporter system will likely lead to more widespread use. While the role of DMT1 presents potential new avenues for MEMRI, it also adds additional variables to the system that must be studied further to clearly understand the extent to which MEMRI measures calcium dynamics from L-type calcium channels separate from other types of ion channels and transporters.

Nearly 30 years following the discovery that manganese impedes calcium dynamics, evidence of intracellular manganese trafficking in vesicles by a microtubule-dependent mechanism was reported in a series of studies (Sloot and Gramsbergen, 1994; Pautler et al., 1998; Takeda et al., 1998). Functionally, this mechanism allows the usage of MEMRI for neuronal tract tracing, a crucial investigative method when considering the methods of manganese administration (discussed later in this section). By nature of being packaged into vesicles similar to neurotransmitters, manganese transports trans-synaptically following fusion of its carrier vesicle with the axon terminal membrane (Serrano et al., 2008). Synaptic manganese is then taken up by the post-synaptic neuron as discussed previously through any of a number of potential calcium-permeable channels or receptors and is then repackaged for further transport propagation. For these mechanistic similarities of manganese and calcium, manganese provides valuable tools for imaging applications but may be limited by substantial toxicity.

### Toxicity

Along with other organ system toxicity, excessive manganese exposure is particularly neurotoxic. These neurotoxic effects include dystonia, impaired speech and poor cognition, and they have been shown to be a particular threat to the developing central nervous system (CNS) throughout childhood (Zoni and Lucchini, 2013; Bjørklund et al., 2017; Lao et al., 2017). Adults are less susceptible to manganese toxicity than children^1^, although neurotoxic (Olanow, 2004; Bowler et al., 2016; Schuh, 2016) and carcinogenic/teratogenic (Gerber et al., 2002) effects have been documented following moderate chronic exposure in adults. Manganism, the classic picture of chronic manganese toxicity in humans, is characterized by motor deficits that closely resemble Parkinson’s disease (PD) in the early stages (Andruska and Racette, 2015). Animal studies have supported the adverse effect findings of chronic manganese exposure. Further neurotoxic potential of manganese is extensively reviewed elsewhere (Chen et al., 2015).

#### Systemic Administration of Manganese

**Figure 2:**
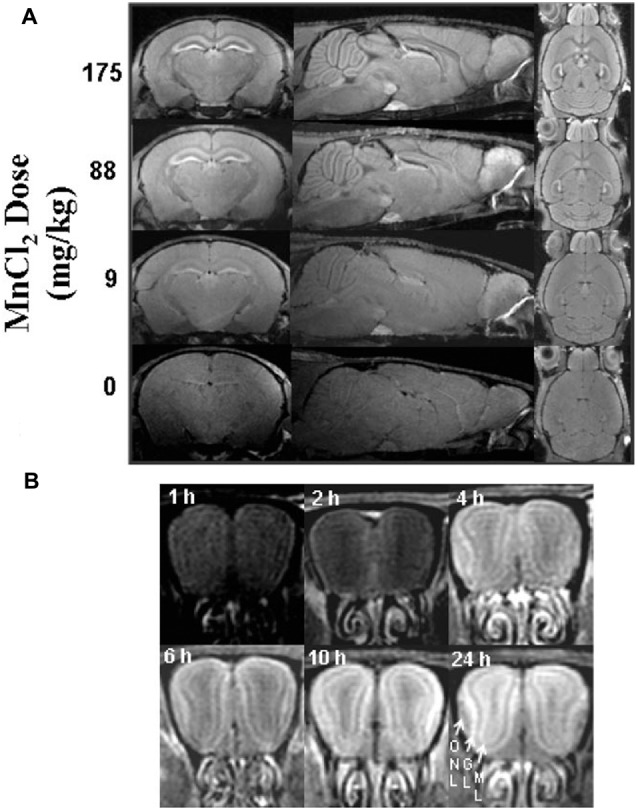
Signal intensity depends on dose and time. Adapted with permission from Lee et al. (2005). Scans performed in mice with intact blood-brain barrier showing the dependence of signal enhancement on **(A)** dose and **(B)** time. **(A)** Manganese produces global enhancement that scales with dose. Enhancement is most significant in the hippocampus, interpeduncular nucleus, pituitary, and olfactory bulb. **(B)** Enhancement of signal increases over time as demonstrated in the olfactory bulb (ONL, olfactory nerve layer; GL, glomerular layer; ML, mitral cell layer).

For imaging studies, manganese solutions are most commonly administered via injections. Koretsky’s early experiments used 25% D-mannitol to break the blood-brain barrier and increase penetration of manganese into the brain (Lin and Koretsky, 1997). Later studies by Koretsky and others determined that MEMRI can be performed in animals with an intact blood-brain barrier, although generally more time and a larger dose of manganese is required to achieve similar enhancement, as demonstrated in Figure 2 (Watanabe et al., 2002; Aoki et al., 2004; Lee et al., 2005; Yu et al., 2005; Kuo et al., 2006). These initial experiments advanced the usage of manganese as a systemically-injected contrast agent for widespread use in imaging.

In the context of MEMRI, the toxicity threshold of manganese remains contested. Since MEMRI studies typically involve a single exposure of moderate to high doses of manganese, these differ from previously described reports on chronic or repeated exposures (Takács et al., 2012; Okada et al., 2016). A study by Eschenko et al. (2010a) looked for signs of toxicity following a single low (0.1 mmol/kg, 16 mg/kg) or high (0.5 mmol/kg, 80 mg/kg) dosage subcutaneous injection of manganese chloride. While the group found no histopathologic differences at either dose, moderate synaptic and motor behavior deficits were observed in rats at the higher dose. Another study by the same group found the synaptic and motor deficits persisted through 1 week following exposure (Eschenko et al., 2010b). These and other studies (Liu et al., 2004; Alaverdashvili et al., 2017) use ranges at or lower than doses typically used for MEMRI, raising concern over potential toxicity and confounding effects of MEMRI.

Other studies found little to no neurotoxicity in mice after single intraperitoneal (IP) doses of manganese chloride at 66 mg/kg (Fontaine et al., 2017), or short-term repeated IP injection in rats reaching final doses of 60 mg/kg (Galosi et al., 2017). To date, many studies have investigated alternative administration paradigms (see next section), alternative manganese-containing compounds (discussed in section “Mangafodipir”) and co-administration of additional compounds (Alahmari et al., 2015; Johnson et al., 2018) to mitigate any potential toxic effects of manganese in MEMRI and still retain useful imaging enhancement.

Fractionated and continuous infusion doses of manganese have been investigated as systemic administration routes that limit toxic effects and exposure for imaging studies. Many studies have noted sufficient manganese enhancement of imaging from fractionated doses, often with no to mild and reversible side effects identified (Bock et al., 2008b; Grünecker et al., 2010; Galosi et al., 2017). One study by Bock et al. (2008a) found fractionated doses of manganese in a non-human primate model has increased longevity of manganese in the brain, notably in the visual cortex and basal ganglia, compared to the rat brain following a similar administration. The authors suggest this species difference may be similar across all mammals, suggesting fractionated dosages may be a viable method in humans using similar manganese-based agents. Similarly, continuous IP infusion of manganese was also found to reduce toxicity relative to a single dose while retaining imaging enhancement (Eschenko et al., 2010a).

Sepúlveda et al. (2012) reported pumps implanted subcutaneously achieve comparable results to fractionated dosing, which allows less invasive implementation of continuous manganese delivery. A more recent study by Vousden et al. (2018) has shown that continuous infusion of manganese via subcutaneous pumps achieves image enhancement without affecting spatial learning or memory. However, this study reported severe and dose-dependent skin ulcerations at the site of implantation in most of the manganese treated mice, whereas control IP injected and saline treated mice did not develop such adverse effects. The authors suggest ulceration may develop due to manganese-induced itching, but this does not sufficiently explain why ulceration has not occurred in more studies investigating subcutaneous manganese pumps. Poole et al. (2017) compared the two methods and found that continuous infusion produced less toxic effects than fractionated injections. However, as this study was published prior to the Vousden et al. (2018) study, it does not consider skin ulcerations. To date, no study has systematically compared fractionated or continuous injection administration of manganese which considers all currently known adverse effects.

**Figure 3:**
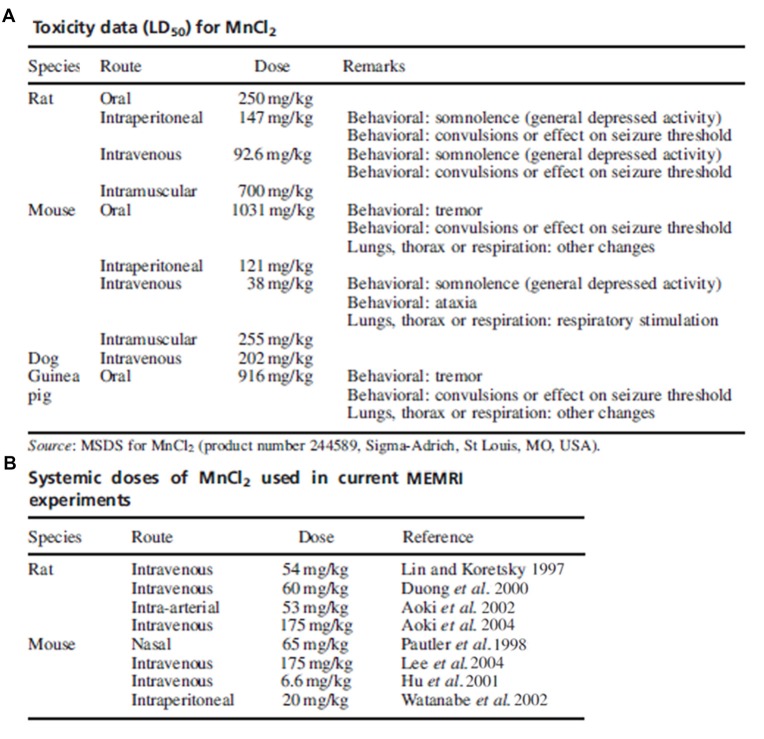
Toxicity data and common doses used in early manganese-enhanced magneticresonance imaging (MEMRI) experiments. Adapted with permission from Silva et al. (2004). **(A)** Summary of toxic manganese doses and associated effects as reported on the MSDS. **(B)** Manganese doses used in several early MEMRI studies in rats and mice. With some exceptions, the dose of manganese used in imaging studies is much lower than the accepted toxic level.

The contention on the toxicity threshold and systemic injection method of manganese highlights the importance in considering previous studies along with the chosen animal model, administration route and dose in determining experimental parameters of manganese for MRI studies. A review from Koretsky’s group demonstrates the variability in dosing and routes of administration used in the first years of modern MEMRI research as summarized in Figure 3 (Silva et al., 2004). If possible, piloting toxicity studies on a per-study basis may provide the only truly sufficient data on toxicity until further investigations reveal consistent thresholds.

#### Localized Administration and Applications

As a viable alternative to systemic routes of manganese administration, a variety of non-systemic administration methods are also successfully used to limit any potential toxic effects. Perhaps the most common non-injection route for manganese exposure is through oral administration. Early studies of oral administration showed sufficient bioavailability of manganese for imaging studies in livers of rats following manganese chloride feeding (Cory et al., 1987). Digested manganese is circulated through and filtered out by the hepatic portal system (i.e., the first past effect), severely reducing systemic distribution of manganese and limiting potential toxicity (Hauser et al., 1994). More recently, oral administration of manganese chloride has been used as an effective and well-tolerated agent for hepatic and hepatobiliary imaging (Leander et al., 2010; Albiin et al., 2012; Marugami et al., 2013). Although manganese removal from the blood is highly efficient, its sensitivity as a contrast agent still facilitates imaging studies in non-privileged body compartments following oral administration (Jacobs et al., 2012). To date, no studies recorded successful MEMRI of CNS structures after oral manganese administration, although manganese reportedly accumulates to levels sufficient to enhance T1 weighted images in patients with cholestatic disease (Ikeda et al., 2000). Given the importance of manganese penetration into the CNS for proper imaging described by Lee et al. (2005), it is still unknown whether oral administration of manganese produces sufficient, safe exposure for clinical MEMRI studies.

One potential method for CNS MEMRI is intranasal administration, which was first reported to deliver manganese to the brains of pike (Tjälve et al., 1995) and rats (Tjälve et al., 1996) sufficient for enhanced imaging. More commonly used today for olfactory imaging studies (Cross et al., 2006; Lehallier et al., 2012b), nasal instillation of manganese reportedly also sufficiently enhances visual cortex imaging in rats (Fa et al., 2010). Though nasal instillation of manganese bypasses the need for systemic administration and may reduce the risk of toxicity, an unintended byproduct is significant nonspecific enhancement (Pautler et al., 1998; Cross et al., 2004). However, later reports suggest this nonspecific enhancement may be reduced with experimental tradeoffs (Chuang and Koretsky, 2009). Additionally, olfactory impairment may occur at doses higher than typically required for imaging (Lehallier et al., 2012a) and moderate inflammation was reported following nasal instillation of manganese solutions (Foster et al., 2018), highlighting potential limitations for its use in CNS MEMRI.

As in the olfactory system, the visual system lends itself to relatively non-invasive methods of manganese administration. Intravitreal injections enhance the retina and visual pathways without the need for systemic administration of manganese. Although intravitreal injection of manganese may result in loss of retinal ganglion cell density at relatively low doses (Thuen et al., 2008), smaller doses provide good enhancement without major signs of damage to retina or other ocular structures (Lindsey et al., 2013).

Topical application of manganese has been investigated as an alternative to intravitreal injection. Topically applied manganese resulted in strong enhancement of ocular structures and the superior colliculus without diffusing into the vitreous space (Sun et al., 2011). The authors posit that the manganese may absorb into the iris and enter the capillary circulation to reach the retina. This hypothesis is supported by the fact that the enhancement was attenuated when retinal ischemia was induced by increasing the intraocular pressure. No adverse changes were observed in the mice 1 week after topical administration of manganese. Similarly, in Sun et al. (2012) the authors administered topical manganese biweekly or monthly in groups of mice. While they found significant retinal ganglion loss and corneal thickening in the biweekly treatment paradigm, no adverse effects were observed when manganese was applied monthly. This was further supported by a later study (Liang et al., 2015) and suggests long-term MEMRI is possible with topical administration of manganese.

**Figure 4:**
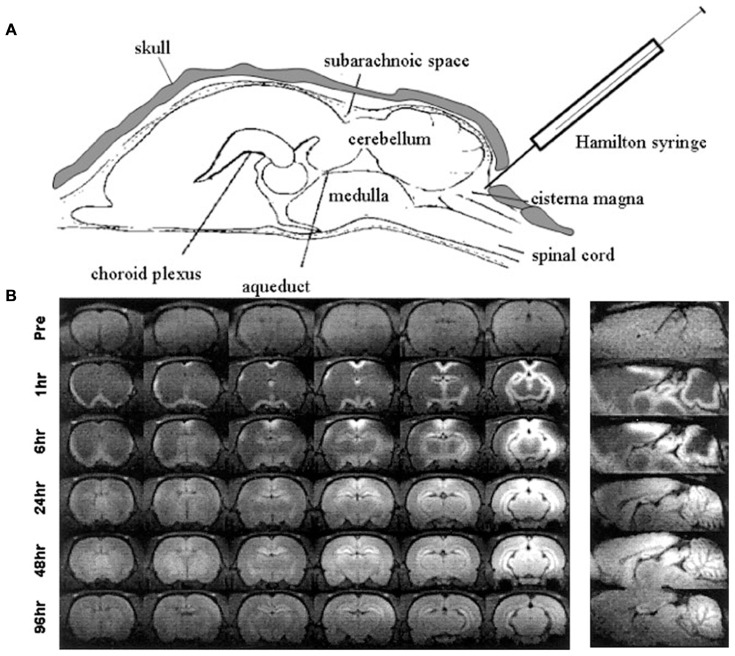
Intrathecal injection of manganese chloride. Adapted with permission from Liu et al. (2004). **(A)** Injection scheme for intrathecal manganese injection. **(B)** Serial images show the spread of enhancement from the ventricles throughout the cortex, subcortex, and cerebellum.

Other methods of administration of manganese by bypassing the blood brain barrier into the CNS have been investigated, stemming from early experiments of injections directly into cerebrospinal fluid (CSF). The earliest application of direct CSF injections involved stereotaxic injection of manganese chloride into the lateral ventricles of rats (Wan et al., 1991). Later, a similar experiment by Liu et al. (2004) achieved measurable enhancement of brain parenchyma 24–96 h following injection of manganese chloride into the cisterna magna (Figure 4). Liu et al. (2004) injected mice with the analogous paramagnetic contrast agent GdDTPA and found no parenchymal enhancement suggesting that the described effect was dependent on cellular uptake of manganese (described in section “Mechanism of Entry and Dispersion of Manganese”). Remarkably, transcranial injection of manganese chloride showed detectable manganese signal in the brain parenchyma within 2 h of administration (Roth et al., 2014). More recently, the Koretsky group expanded upon this technique by showing that manganese penetrates into underlying brain structures when applied transcranially by passing through brain suture lines (Atanasijevic et al., 2017). While transcranial application of manganese for MEMRI requires further optimization before widespread use, its potential in relatively noninvasive MEMRI studies are becoming extremely valuable.

#### Effect of Blood Brain Barrier Permeability

Poor blood-brain barrier permeability has been a major obstacle in MEMRI studies of the CNS. Several MEMRI protocols call for chemical (Lin and Koretsky, 1997; Lu et al., 2010) or mechanical (Howles et al., 2012) disruption of the blood-brain barrier to improve penetration of manganese into the CNS. The integrity of the blood-brain barrier has a significant effect on the penetrance of manganese into the CNS, and because of this several studies have used manganese to evaluate changes in blood-brain barrier permeability (Fitsanakis et al., 2006; Grillon et al., 2008; Nischwitz et al., 2008). It may be advisable to evaluate animals for blood-brain barrier damage to eliminate potential confounding variables that could arise from differential penetrance of manganese into the CNS as this could result in apparent differences in MEMRI signal. Furthermore, animals with possible blood-brain barrier dysfunction should be monitored more closely for signs of manganese-related injury as they are more likely to reach toxic accumulation of manganese in the brain.

## MEMRI in CNS Imaging

One major application of MEMRI is functional imaging of the CNS. The technique has been applied to a variety of CNS pathologies including traumatic brain injury, epilepsy, neurodegeneration and pain. Additionally, MEMRI has been used heavily in studies of the olfactory and visual systems. These areas of study are by no means mutually exclusive, and recurrent patterns will emerge between areas of MEMRI implementation that may suggest future avenues for investigation.

### Traumatic Brain Injury

Traumatic brain injury (TBI) is a serious threat to health, contributing to 30% of all injury related deaths in the United States according to the CDC^2^. Glutamate increases sharply in animal models following acute TBI (Palmer et al., 1993), a finding that’s been supported in human studies (Brown et al., 1998; Yamamoto et al., 1999; Ruppel et al., 2001). Excitotoxicity leads to activation of voltage-gated calcium channels, increasing intracellular calcium concentration (Young, 1992). High intracellular calcium concentrations play a significant role in cell injury and death (Trump and Berezesky, 1995). As discussed previously, manganese influx can occur concurrently with calcium influx, which allows MEMRI to monitor changes in calcium dynamics after TBI.

The first study to apply MEMRI to TBI measured changes after diffuse TBI in rats (Cernak et al., 2004). Subsequent studies found varying patterns of signal enhancement following TBI, which is potentially due to disturbances in the blood-brain barrier (Bouilleret et al., 2009; Rodriguez et al., 2016). Talley Watts et al. (2015) found that manganese-enhanced images showed crescent-shaped areas of hyperintensity at the impact site corresponding to areas of reactive gliosis, a finding that was further supported by positive GFAP staining. Comparisons between these studies are difficult due to inherent differences in the particular models of TBI employed, but despite these differences, MEMRI is effective to measure changes in brain function after injury. Of particular note, one study by Tang et al. (2011) used MEMRI to successfully track migration and function of human neural stem cells implanted in rats after TBI. They went on to show that this activity was attenuated by treatment with the calcium channel blocker diltiazem, which supports the findings of Lu et al. (2007) discussed previously.

### Epilepsy

Epilepsy is a neurological condition characterized by recurrent seizures. It is estimated to affect 50 million people worldwide^3^. In humans, temporal lobe epilepsy (TLE) is the most common type of focal epilepsy (Asadi-Pooya et al., 2017). Status epilepticus (SE), defined as a seizure lasting more than 30 min, is a medical emergency that can result in significant morbidity and mortality (Cherian and Thomas, 2009). Currently, electroencephalogram (EEG) is the most commonly used modality for monitoring epilepsy, and MRI plays a crucial role during diagnosis (Rüber et al., 2018).

One of the most consistent features of TLE and SE in human and animal models is mossy fiber sprouting in the dentate gyrus of the hippocampus beginning in the first week following epileptogenesis and continuing to develop for months after (Mathern et al., 1995; Smith and Dudek, 2001; Scharfman et al., 2003; Shetty et al., 2003). Nairismägi et al. (2006) showed *in vivo* MEMRI evidence of mossy fiber sprouting following drug-induced SE in rats, which was later confirmed via histopathology. This finding has since been replicated in multiple models of TLE and SE (Immonen et al., 2008; Malheiros et al., 2012) and studies have used MEMRI to detect focal edema, neuronal death and astrocyte proliferation in the hippocampus of rats as a result of sustained seizure activity (Hsu et al., 2007; Malheiros et al., 2014). One study found a negative correlation between hippocampus signal intensity and seizure frequency, suggesting a role for MEMRI in preclinical assessment of epileptogenesis severity in future studies (Dedeurwaerdere et al., 2013).

Sudden unexplained death in epilepsy (SUDEP) is a major concern for people with epilepsy, and it accounts for approximately 15% of epilepsy related deaths (Tomson et al., 2016). As the name suggests, SUDEP is difficult to predict although seizure frequency is positively correlated to risk. Recently, MEMRI was used to show changes in an audiogenic seizure mouse model consistent with human SUDEP (Kommajosyula et al., 2017). This model develops tonic seizures leading to respiratory arrest that is fatal without resuscitation. MEMRI performed during seizure-induced respiratory arrest showed increased signal intensity in regions of the superior colliculus, periaqueductal gray and amygdala, brain regions previously implicated in SUDEP in humans (Mueller et al., 2014; Tang et al., 2014; Wandschneider et al., 2015). Future studies will be needed to better adapt MEMRI to the study of SUDEP, but continued efforts may provide better risk stratification and preventative measures.

### Neurodegeneration

Neurodegenerative diseases are a debilitating class of conditions involving progressive brain atrophy and loss of cognitive and/or motor function. This class comprises tauopathies (including Alzheimer’s disease (AD) and frontotemporal dementia), PD, Lewy body disease, amyotrophic lateral sclerosis (ALS) and Huntington’s disease. Despite years of ongoing research, the prognosis for patients diagnosed with these conditions is generally poor. To date, studies have explored the role of MEMRI in context of tauopathies, PD and ALS. No studies are currently available describing the use of MEMRI to investigate Huntington’s disease or Lewy body disease; however, given the relative youth of the field and the rapid expansion over the past two decades, future research may find utility of MEMRI in studying these conditions.

#### Alzheimer’s Disease and Other Tauopathies

AD, the most common cause of dementia, is part of the class of related diseases termed tauopathies (Bertram and Tanzi, 2005). These diseases vary widely in geographic involvement and symptomatic presentation, but all share underlying tau pathology as a basis for neurodegeneration. Tau protein is classically involved with stabilizing microtubules and loss of tau function mediates axonal degeneration in many tauopathy cases (Kneynsberg et al., 2017). Confirmed diagnoses for tauopathies cannot be made until post-mortem examination confirms histopathology. This major obstacle in the diagnosis of tauopathies compounds with the problem that appropriate therapies for one type of tauopathy likely will not be effective for another (Coughlin and Irwin, 2017), establishing the importance of identifying the tauopathy as early as possible.

The first application of MEMRI for research into tauopathies quantified differences in axonal transport (Smith et al., 2007). In this study, it was shown that MEMRI could detect decreased rates of axonal transport in the Swedish mutant APP mouse, a model of AD characterized by secondary tau pathology. Many other studies have since used MEMRI to show impairments or therapy-related improvements in axonal transport in mouse models of AD or tauopathies (Massaad et al., 2010; Smith et al., 2010, 2011; Gallagher et al., 2012; Wang et al., 2012; Majid et al., 2015; Saar et al., 2015). MEMRI has also been used to demonstrate axonal deficits in the olfactory pathways of tau-transgenic JNPL3 (Bertrand et al., 2013) and rTg4510 (Majid et al., 2014) mouse models. Further supporting these findings, Fontaine et al. (2017) showed broad changes in neuronal function in preclinical rTg4510 mice following systemic administration of manganese. With detectable changes in the asymptomatic stage of the disease, these studies highlight the potential application of MEMRI in preclinical identification of tau pathology *in vivo*.

#### Parkinson’s Disease

PD is the second most common neurodegenerative condition (Bertram and Tanzi, 2005) and involves the progressive loss of dopaminergic neurons in the substantia nigra leading to a characteristic pattern of impaired movement (Hughes et al., 1992). Neurological manifestations of PD include cognitive impairment, impulse control disorders and circadian rhythm dysfunction (Mantovani et al., 2018; Marques et al., 2018; Weil et al., 2018; Weintraub et al., 2018).

Initiation and control of movement relies on close communication between the basal ganglia and substantia nigra (Lanciego et al., 2012). Manganese deposits in the basal ganglia (Nelson et al., 1993; Fredstrom et al., 1995; Nagatomo et al., 1999), and this is the basis for motor deficits associated with manganese toxicity as previously discussed and may explain the relative paucity of studies employing MEMRI to investigate PD.

The earliest study to use MEMRI in the context of PD supported previous observations that interhemispheric cortical connectivity observed in humans and rats is mediated through the basal ganglia (Pelled et al., 2007). Direct injection of manganese chloride into the subthalamic nucleus in rat of the 6-hydroxydopmaine model of PD reveals impaired transport of manganese throughout the basal ganglia-substantia nigra circuit indicating impaired axonal transport (Soria et al., 2011). In addition to establishing connectivity between brain regions in PD, two recent studies highlighted the potential for MEMRI in monitoring response to novel therapeutics (Olson et al., 2016; Weng et al., 2016).

#### Amyotrophic Lateral Sclerosis

ALS is characterized by progressive degeneration of upper (cortical) and lower (spinal) motor neurons leading to generalized weakness (Peters and Brown, 2015). Patients gradually become weaker and succumb to respiratory failure. The current standard therapy for ALS is riluzole, which appears to slow progression of the disease, as well as physical and speech therapy and respiratory support^4^. While the exact etiology of ALS is currently unknown, deficits in axonal transport have been identified (Collard et al., 1995).

To date, only one study could be found which applied MEMRI to ALS (Jouroukhin et al., 2013). In this study, davunetide was shown to slow disease progression in a mouse model of ALS, thereby increasing the speed of axonal transport and protecting against neuronal loss. Davunetide functions by stabilizing microtubules, thereby preventing colchicine-mediated degradation (Jouroukhin et al., 2013; Magen and Gozes, 2013). Interestingly, davunetide was previously evaluated for therapeutic effects in the tauopathy progressive supranacular palsy, although it ultimately proved to be ineffective for this use. Given the common involvement of axonal deficits and the overlap between therapeutic approaches, one could expect more studies to employ MEMRI in the context of ALS in the future.

### Pain

Chronic pain is a complex condition that causes significant loss of quality of life in approximately 7%–8% of adults worldwide^5^. Neuropathic pain, which arises from damage to the somatosensory system, takes many forms including central neuropathic pain, polyneuropathy, post-amputation pain and HIV-associated neuropathy. For many years, opioid analgesics have been the standard therapy for chronic pain. Given the current rates of opioid abuse facing the United States, there is a significant effort to develop alternate approaches to treat chronic pain (Downes et al., 2018; Morad et al., 2018). Furthermore, pain is known to adversely affect the mental health of affected patients (Goesling et al., 2018). One aspect of chronic pain that complicates development of a comprehensive therapeutic approach is that many of the pathways underlying the development and maintenance of pain are not well understood.

The earliest studies to use MEMRI in a pain-related setting examined the use of acupuncture for analgesia (Chiu et al., 2001). After showing changes in brain activity after acupuncture via MEMRI, Chiu et al. (2001) compared activation patterns between electroacupuncture at points associated with analgesic or non-analgesic properties (Chiu et al., 2003). While acupuncture at either site was associated with activity in the somatosensory cortex and hypothalamus, acupuncture at the analgesic site also increased activation in the periaqueductal gray and median raphe nucleus; these regions specifically involved in the processing of pain. This study was the first to demonstrate the use of MEMRI in identifying pain pathways.

Later, Yang et al. (2011) published the first report to use MEMRI to study pain specifically. After injecting manganese chloride into the thalamus, electrical current was applied to the forepaw of a rat to induce pain. Subsequent imaging showed the strong activation in the anterior cingulate and midcingulate cortex, areas that were previously well-established in pain processing. This study also identified the ventral medial caudate-putamen and nucleus accumbens as possible components of pain processing circuitry. Pain-induced activation in each of these areas was attenuated by pretreatment with morphine. Recently, Sperry et al. (2017) performed similar imaging in perfused brains, allowing much longer scan times to improve resolution.

Since Yang et al. (2011) first used MEMRI to map pain circuits, the technique has been used in several studies of pain. MEMRI has proved effective for studying irritant injection (Devonshire et al., 2017; Sperry et al., 2017), nerve injury (Behera et al., 2013; Jeong and Kang, 2018) and thermal (Lei et al., 2014) models of pain. Interestingly, in an investigation into the difference between processing of neuropathic pain and pathological itching using MEMRI, Jeong et al. (2016) found differences in processing for each stimulus in the limbic systems. However, further studies are needed to better describe this process.

### Olfactory System

MEMRI studies have long been employed for use in studying the olfactory system, owing largely to the ease with which manganese can be applied via the nasal mucosa. The first study to demonstrate tract tracing via MEMRI were performed in the olfactory bulb after nasal instillation of manganese (Pautler et al., 1998). As previously described, tract tracing requires introduction of manganese solutions to specific regions of the CNS, typically via intracranial or intravitreal injection. The olfactory receptors in the nasal mucosa project directly to the olfactory bulb, allowing tract tracing in this region with a less invasive route of administration. Pautler and Koretsky (2002) later showed region specific activation in the mouse olfactory bulb in response to aerosolized urine odorants. Subsequent work used the rodent olfactory system to refine the process of tract tracing (Lehallier et al., 2011).

Since these early reports many additional reports have used MEMRI to map circuits in rodent olfactory systems. Chen et al. (2007) observed differences in activation patterns between unconditioned arousal (lemon) and fear (fox) odorant stimuli. Work from the Koretsky lab found specific activation patterns in the olfactory bulb corresponding to different odorants achieving resolution of individual glomerular cells (Chuang et al., 2009, 2010). Gutman et al. (2013) combined MEMRI with DTI and found the imaging modalities compatible and complementary for the purpose of tracing neural circuits.

MEMRI studies of the olfactory system have also been used in disease-specific context. Several studies showed deficits in axonal transport in the olfactory bulb of neurodegenerative mice, as previously described in the context of tauopathy (Smith et al., 2007, 2010, 2011; Wang et al., 2012; Bertrand et al., 2013; Majid et al., 2014; Saar et al., 2015) and ALS (Jouroukhin et al., 2013). MEMRI has similarly shown changes in olfactory function in animal models of cerebral palsy (Drobyshevsky et al., 2006, 2012), neuropsychiatric lupus (Kivity et al., 2010) and diabetes (Sharma et al., 2010). Gobbo et al. (2012) used MEMRI to study the effects of glutamate excitotoxicity in the olfactory bulb, modeling a possible mechanism of atrophy associated with diseases such as AD or stroke. These studies demonstrate the utility of MEMRI to detect neuronal changes in the olfactory bulb that may represent disease specific processes or broader changes in neuronal function.

### Visual System

**Figure 5:**
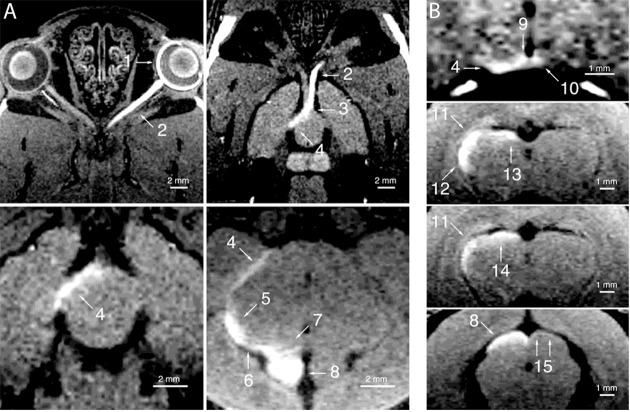
Manganese enhanced tracing of the rat visual system. Adapted with permission from Watanabe et al. (2001). Enhancement of the visual pathway 24 h after intravitreal injection of manganese. Images were collected in the **(A)** horizontal and **(B)** coronal planes. 1 = left retina, 2 = left optic nerve, 3 = optic chiasm, 4 = right optic tract, 5 = right lateral geniculate nucleus, 6 = right brachium of the superior colliculus, 7 = right pretectal region, 8 = right superior colliculus, 9 = right suprachiasmatic nucleus, 10 = left suprachiasmatic nucleus, 11 = right dorsal geniculate nucleus, 12 = right ventral lateral geniculate nucleus, 13 = right olivary pretectal nucleus, 14 = right nucleus of the optic tract, 15 = superficial part of the superficial gray layer of the left superior colliculus.

As discussed previously (section “Localized Administration and Applications”), the visual system lends itself to relatively noninvasive methods of manganese administration. Historically, MEMRI was first used to study the mapping of the visual pathway from retina to superior colliculus (Watanabe et al., 2001; Figure 5). With this study supporting the theoretical role of MEMRI in visual system further investigations have delved into the structure, function and tractographical details and diseases in a variety of animal models.

#### Mapping the Visual System

Following successful mapping of the visual system by Watanabe et al. (2001), studies used manganese to show finely-tuned changes in functional differences of the visual system. For example, Bissig and Berkowitz (2009) showed systemic manganese administration and visual stimulation revealed discrete layer-specific changes in function in the visual cortex of rats.

Subsequent research by Chan et al. (2011b, 2014) and Chan and Wu (2012) expanded these previous studies to assess neuroarchitecture and functional relationships in the rat visual system using a variety of manganese injection techniques. They first investigated visual system development and found faster axonal transport of manganese in the developing rats, attributed to higher permeability of the blood-ocular and blood-brain barriers in the immature rat (Chan et al., 2011a). With an increase in detectable projections from both the retina and visual cortex following enucleation, this study demonstrates the ability of MEMRI to not only map the visual system, but also to detect finer neuroplastic changes. Other studies have since demonstrated the ability of MEMRI to detect sensory system-wide neuroplastic changes (Tang et al., 2017a,b).

Chan et al. (2014) conducted additional experiments to more fully characterize the normally functioning rat visual system. They partially transected the optic nerve near the optic head to show retinotopic attenuation of signal in the superior colliculus. Later studies further expanded the connectivity work previously performed, using varied injection techniques (intravitreal, intracortical, subcortical) to provide more detailed descriptions of the connections between parts of the visual system (Chan and Wu, 2012).

#### Retinal Structure and Function

Another area of vision-related research that has benefitted greatly from application of MEMRI is the study of retinal function. The first application of MEMRI to the retina measured differences in ion demand between light- and dark-adapted rats (Berkowitz et al., 2006), an application which has since been replicated (De La Garza et al., 2012). Subsequent studies from Berkowitz and colleagues provided *in vivo* descriptions of ion regulation through the visual cycle (Berkowitz et al., 2009b), activity of channelrhodopsin-2 (Ivanova et al., 2010) and horizontal cell inhibitory signaling (Berkowitz et al., 2015b). These experiments established MEMRI as a sensitive technique capable of producing *in vivo* resolution of retinal layers to establish biochemical understandings.

Additional studies have demonstrated that MEMRI can be used to study degenerative pathology associated with the retina. Berkowitz and colleagues used MEMRI to show changes in retinal ion demand in models of ocular injury (Berkowitz et al., 2007a), retinopathy of prematurity (Berkowitz et al., 2007b) and retinal thinning (Berkowitz et al., 2008). Nair et al. (2011) showed layer resolution and lamina-specific structures in degenerating rat retina, which highlighted the potential for disease monitoring via MEMRI. This potential was further expanded when another group used MEMRI to show the effects of prophylactic retinylamine therapy in a mouse model of retinal degeneration (Schur et al., 2015).

#### Optic Nerve Injury and Regeneration

In addition to investigating the retina, MEMRI can be applied to study injury and regeneration of the optic nerve. The capacity for MEMRI studies to detect injury-related changes in optic nerve function has been well-established (Ryu et al., 2002; Thuen et al., 2005) and MEMRI can be used in conjunction with DTI to provide more detailed evaluation (Thuen et al., 2009). Work from Sandvig et al. (2011) used MEMRI to monitor optic nerve regeneration in four different animal models longitudinally. Shortly after, they showed evidence that transplanted olfactory ensheathing cells mediate repair and remyelination in damaged optic nerves (Sandvig et al., 2012). Additional studies have further demonstrated the use of MEMRI in assessing optic nerve injury and repair (Haenold et al., 2012; Fischer et al., 2014; Yang et al., 2016).

#### Diabetic Retinopathy

Diabetes is a chronic, systemic condition characterized by persistent high blood sugar associated with a variety of negative conditions including heart disease, stroke, kidney failure, peripheral neuropathy and impaired vision or blindness^6^. Ocular manifestations of diabetes, particularly diabetic retinopathy, are a leading cause of visual impairment and preventable blindness worldwide (Lee et al., 2015). Diabetic retinopathy can be detected reliably via fundoscopic examination; however, due to the asymptomatic early stages and limited ophthalmologic care in developing nations, many cases remain undiagnosed until permanent damage has occurred (Viswanath and McGavin, 2003).

MEMRI has been suggested as a viable method to study the processes associated with the development of diabetic retinopathy and to monitor therapeutic responses. For example, manganese-enhanced imaging shows *in vivo* ion dysregulation (Berkowitz et al., 2009a) and oxidative stress (Berkowitz et al., 2015a) in diabetic mice, providing potential mechanistic insight into the disease. Furthermore, MEMRI detects changes in retinal function 14 days after induction of hyperglycemia, earlier than any previous time point in literature (Muir et al., 2015). In addition to studying disease progression, MEMRI has been used to assess several potential therapeutic approaches for diabetic retinopathy (Berkowitz et al., 2007c, 2012; Giordano et al., 2015).

#### Glaucoma

Glaucoma is a group of related diseases that result in abnormally high intraocular pressure. Left untreated, the high pressure can damage the optic nerve, leading to permanent impairment or loss of vision^7^. Like diabetic retinopathy, glaucoma is a major cause of vision loss worldwide (Tham et al., 2014). The prevalence of glaucoma increases with age and the number of people affected by glaucoma is projected to double by 2040. Therefore, continued research is necessary to adapt to the increasing health challenges faced by an increasingly aged population.

Though limited in number, the studies employing MEMRI nevertheless demonstrate a role for MEMRI in assessing glaucoma pathology. Studies using MEMRI identified impaired axonal transport in glaucomatous eyes of rats compared to normal prior to the development of changes in retinal thickness (Chan et al., 2007, 2008; Calkins et al., 2008). Data collected via MEMRI suggest the development of glaucoma may be more complicated than previously thought (Fiedorowicz et al., 2018). Therefore, more research in this field will be required to better understand progression of the disease as well as the optimal methods to study it *in vivo*.

### Auditory System

The first studies to use MEMRI in the study of the auditory system came from the Turnbull group. They generated the first tonotopic map of the inferior colliculus, showing functional changes associated with varying degrees of hearing loss (Yu et al., 2005). Subsequent studies applied MEMRI to describe development and plasticity of the auditory system (Yu et al., 2007) and to examine the effect of frequency and amplitude on auditory processing in the inferior colliculus (Yu et al., 2008). In these studies, manganese was administered to mice immediately before a sound exposure experiment. Because neuronal activity correlates with manganese uptake, this paradigm allows for brain responses to be encoded away from the noisy environment of the MRI scanner. Manganese in the stimulated brainstem regions persisted long enough to allow the activation pattern to be measured 24 h later.

MEMRI studies of the auditory system can be performed following intratympanic injection of manganese chloride. Analogous to the tracing of the visual pathways performed by Thuen et al. (2005), intratympanic administration of manganese produces sequential enhancement of the auditory system from cochlea to inferior colliculus (Lee et al., 2012). Subsequent work found that auditory pathway tracing is sensitive to changes in the frequency and amplitude of the sound stimulus (Jin et al., 2013) and this mapping technique had been applied to disease models (Jung et al., 2014).

In addition to mapping the auditory system, MEMRI has been used to study auditory disorders including hearing loss and tinnitus. Using MEMRI, Gröschel et al. (2011) identified changes in calcium-dependent activity in the central auditory system associated with noise-induced, age-related (Gröschel et al., 2014) and drug-induced hearing loss (Gröschel et al., 2016), thereby providing novel insights into these conditions and suggesting that multiple mechanisms may produce similar symptoms across different modalities of hearing loss. MEMRI studies have also demonstrated abnormal neuronal function in animal models of tinnitus. Brozoski et al. (2007) measured hyperactivity in brain regions including the cochlear nucleus, inferior colliculus, cerebellar paraflocculus and amygdala. This study was the first to identify abnormal cerebellar function associated with tinnitus. A follow-up study attributed the tinnitus-related hyperactivity to abnormal NMDA activity, demonstrating the NMDA blockade improves symptoms (Brozoski et al., 2013). These studies from Brozoski et al. (2007, 2013) described a previously unidentified interaction between the paraflocculus and cochlear nucleus as a necessary component of noise-induced tinnitus. Subsequent work has expanded these findings to include drug induced models of tinnitus and implicated additional brain regions in the pathology (Holt et al., 2010; Muca et al., 2018). Consistent with previous work, these studies strongly implicate brain stem structures (particularly the inferior colliculus) in the development of tinnitus and found no tinnitus-related changes in function in the auditory cortex.

## Mangafodipir

Chelated manganese compounds such as mangafodipir (MnDPDP, Teslascan) provide an alternative to potentially toxic manganese chloride solutions for use in clinical applications of MEMRI. Mangafodipir is prepared by chelating ionic manganese with the organic ligand fodipir (Rocklage et al., 1989) producing a complex metabolized in humans to release manganese ions for enhancement in MR imaging studies (Toft et al., 1997a,b). Mangafodipir was first used to show ischemia associated with myocardial infarctions (Pomeroy et al., 1989; Saeed et al., 1989), but its primary usage has been as a contrast for hepatobiliary imaging (Rofsky and Weinreb, 1992). Its use expanded considerably since FDA approval in 1997.

### Animal Studies With Mangafodipir

Quickly following its original intended use, mangafodipir substantially enhanced hepatobiliary imaging without significant toxicity. Studies in rats evaluating its toxicity for MEMRI reported toxicity at high doses but with a high therapeutic index (Elizondo et al., 1991). A later study showed that mangafodipir was not associated with injection site or dermal hypersensitivity reactions (Larsen and Grant, 1997), which is in contrast to later studies of manganese injections. The potential negative ionotropic effects of manganese in the heart were balanced by a release of catecholamines triggered by MnDPDP *in vivo* (Jynge et al., 1997). Furthermore, unlike manganese chloride, mangafodipir does not cause higher levels of manganese accumulation in the brain in animals with biliary obstruction compared to control (Grant et al., 1997b). It should be noted, however, that mangafodipir induced skeletal abnormalities in fetal rats, suggesting teratogenicity (Grant et al., 1997a).

For the purposes of imaging, the major differences observed between MEMRI studies with manganese chloride and mangafodipir is the time to maximal enhancement. The slow release of manganese during mangafodipir metabolism compared to solutions of manganese chloride produces a more gradual rise in manganese concentration (Ni et al., 1997) with no loss of enhancement (Southon et al., 2016). A later study of retinal function after systemic mangafodipir administration and MEMRI detected changes in retinal function consistent with previous studies done with manganese chloride (Tofts et al., 2010).

### Human Studies With Mangafodipir

The use of mangafodipir in human MRI studies began shortly following successful animal imaging studies and focused on tumor and lesion identification in the hepatobiliary system. The first use of mangafodipir MEMRI in human subjects demonstrated enhancement of the liver parenchyma within 15 min of intravenous injection without major adverse effects (Lim et al., 1991). The most commonly reported effect of mangafodipir injection is facial flushing and warmth and minor adverse events including nausea, headache, elevated blood pressure and accelerated heart rate (Lim et al., 1991; Wang et al., 1997b).

Several stage II clinical trials and other studies have shown mangafodipir-enhanced MRI to be effective for identifying tumors and metastases in the human hepatobiliary system (Bernardino et al., 1991, 1992; Rummeny et al., 1991, 1997; Hamm et al., 1992; Wang et al., 1997a). The sensitivity of mangafodipir enhanced MRI is highest for tumors or hepatocellular origin (Aicher et al., 1993; Rofsky et al., 1993; Vogl et al., 1993).

Following these successful studies, several stage III clinical trials compared mangafodipir enhanced MRI with human-approved contrast agents and found that it improved identification of hepatocellular carcinoma (Kettritz et al., 1996) and detection of focal lesions (Diehl et al., 1999) over gadolinium based contrasts, but no difference was found in the ability to detect liver metastases or other masses (Kettritz et al., 1996; Schima et al., 1997).

Other studies have compared mangafodipir-enhanced MRI with other methods of clinical imaging modalities such as computed tomography (CT). Several have reported greater efficacy of mangafodipir enhanced MRI to contrast enhanced CT imaging for detection of hepatocellular lesions (Bartolozzi et al., 2000; Federle et al., 2000; Oudkerk et al., 2002). Mangafodipir-enhanced MRI has additionally shown similar accuracy for diagnosis and staging of pancreatic cancer compared to contrast enhanced CT, but neither modality demonstrated a clear advantage (Rieber et al., 2000; Romijn et al., 2000).

### Non-imaging Uses of Mangafodipir

Despite these promising clinical trials, mangafodipir was removed from the European market in 2012^8^ due to poor sales and is similarly listed as discontinued by the FDA^9^. We found limited evidence of non-marketing related reasons behind these regulatory decisions. However, research using mangafodipir has been ongoing. Two metabolites of mangafodipir, MnPLED and ZnPLED, have exhibit antioxidant properties through actions mimicking superoxide dismutase (SOD) in rats (Brurok et al., 1999). When donor rats were pretreated with MnDPDP before liver transplant, the recipient experienced reduced ischemic injury after transplantation (Ben Mosbah et al., 2012). Later studies in humans reported mangafodipir administration reduces cardiac injury associated with chemotherapy (Yri et al., 2009) and post-myocardial infarction reperfusion (Karlsson et al., 2015).

Recently, the efficacy of mangafodipir as an adjunct to chemotherapy has been established. In culture, co-administration of mangafodipir with the anti-cancer drugs oxaliplatin or 5-fluorouracil resulted in increased killing of mouse colon cancer cells and improved survival of human leukocytes *ex vivo* (Alexandre et al., 2006). The mechanisms of this differential targeting are unclear, but the authors speculate that the increased oxidative stress at baseline in the cancer cells compared to normal is a contributing factor. A preliminary trial in human subjects similarly preserved leukocyte counts during treatment with oxaliplatin and 5-fluorouracil (Karlsson et al., 2012a). Calmangafodipir, a derivative complex of mangafodipir in which some of the manganese is replaced by calcium, exhibits a greater degree of myelo-preservation while still enhancing antitumor effects (Karlsson et al., 2012b). Two additional studies show that mangafodipir reduces the occurrence of oxaliplatin-induced peripheral neuropathy in human patients (Coriat et al., 2014; Karlsson et al., 2017) and is an active area of interest. Future studies are needed to better characterize how mangafodipir and related compounds interact with anti-cancer therapies, but current research shows promise.

## Conclusion

Manganese provides useful enhancement of MR images by nature of its paramagnetic properties. Augmented by having cell permeability like that of calcium, manganese application for MRI provides unique functional imaging capacities. Over the last 40 years, research using applied MEMRI has delved into the structure, function and tractography in a wide variety of investigative areas. In the CNS, the functional component of MEMRI provides unique insight into the cellular mechanisms of brain disorders and neurodegenerative diseases like AD. Concerns over the toxicity and administrative methods of manganese *in vivo* have spurred the use of manganese-chelated compounds such as mangafodipir for MEMRI clinically, though no recorded studies have reported uses in human CNS imaging. Current applications show renewed promise of manganese- and chelated MEMRI usage for research questions.

## Author Contributions

RC, SK and JA wrote and edited the manuscript.

## Conflict of Interest Statement

The authors declare that the research was conducted in the absence of any commercial or financial relationships that could be construed as a potential conflict of interest. The handling Editor declared a shared affiliation, though no other collaboration, with the authors.

## Footnotes

^1^Agency for Toxic Substances and Disease Registry, *Manganese*, https://www.atsdr.cdc.gov/toxprofiles/tp151-c2.pdf

^2^Centers for Disease Control and Prevention, TBI: Get the Stats on Traumatic Brain Injury in the United States, https://www.cdc.gov/traumaticbraininjury/get_the_facts.html [Accessed March 12, 2018].

^3^World Health Organization,* Epilepsy Fact Sheet*. http://www.who.int/news-room/fact-sheets/detail/epilepsy [Accessed March 12, 2018].

^4^National Institute of Neurological Disorders and Stroke, *Amyotrophic Lateral Sclerosis (ALS) Fact Sheet*. https://www.ninds.nih.gov/Disorders/Patient-Caregiver-Education/Fact-Sheets/Amyotrophic-Lateral-Sclerosis-ALS-Fact-Sheet [Accessed March 13, 2018].

^5^International Association for the Study of Pain. Fact Sheets. https://www.iasp-pain.org/Advocacy/Content.aspx?ItemNumber=3934 [Accessed March 14, 2018].

^6^Centers for Disease Control and Preventions, *At a Glance 2016: Diabetes*. https://www.cdc.gov/diabetes/library/factsheets.html [Accessed March 15, 2018].

^7^National Eye Institute, *Facts About Glaucoma*. https://nei.nih.gov/health/glaucoma/glaucoma_facts [Accessed March 16, 2018].

^8^European Medicines Agency, *Public statement on Mangafodipir*, EMA/486286/2012 (London, UK, 2012)

^9^U.S. Food and Drug Administration, FDA approved drug products: Teslascan. https://www.accessdata.fda.gov/scripts/cder/daf/index.cfm?event=overview.process&ApplNo=020652. [Accessed March 22, 2018].
